# Population genomic analysis reveals genetic divergence and adaptation in *Brachymystax lenok*

**DOI:** 10.3389/fgene.2024.1293477

**Published:** 2024-02-28

**Authors:** Ping Li, Le Niu, Jianbo Chang, Xiaomei Kou, Wentian Wang, Wenjing Hu, Qigen Liu

**Affiliations:** ^1^ Powerchina Northwest Engineering Corporation Limited, Xi’an, China; ^2^ State Key Laboratory of Water Resources and Hydropower Engineering Science, Institute of HydroEcology, Wuhan University, Wuhan, China; ^3^ Hubei Key Laboratory of Water System Science for Sponge City Construction, Wuhan, China; ^4^ Hanjiang-to-Weihe River Valley Water Diversion Project Construction Co., Ltd., Xi’an, China; ^5^ Centre for Research on Environmental Ecology and Fish Nutrition, Ministry of Agriculture and Rural Affairs, Shanghai Ocean University, Shanghai, China; ^6^ Key Laboratory of Integrated Rice-fish Farming, Ministry of Agriculture and Rural Affairs, Shanghai Ocean University, Shanghai, China

**Keywords:** population genomics, genome divergence, ecological adaptation, Brachymystax lenok, SNP

## Abstract

Studying how populations in various environments differ genetically is crucial for gaining insights into the evolution of biodiversity. In order to pinpoint potential indicators of divergence and adaptation to diverse environments, we conducted a comprehensive analysis of 3,491,868 single nucleotide polymorphisms (SNPs) derived from five populations of *Brachymystax lenok*. We discovered significant geographic divergence among these 5 populations, which lack evidence of gene flow among them. Our results further demonstrated that the current distribution pattern of *Brachymystax lenok* are driven by geographical isolation and changes in oceans and rivers. We also performed genome-wide scan and identified the genes evolved to adapt the different environments, including stress response. In general, these results provide genomic support for high-level genetic divergence and the genetic basis of adaptation to different environments.

## Introduction

Understanding the evolutionary processes driving the evolution of biodiversity is one of the central goals in evolutionary biology. Earlier studies have thought geographical features and palaeoclimatic oscillations are common driving forces for such processes (Li et al., 2023). For example, it has been considered that mountains and rivers are barriers to drive vicariance across many organisms (Zhang et al., 2005; Zhang et al., 2006; Che et al., 2010). This process is often observed among lineages distributed on different sides of mountains and rivers with deep genetic divergence (Yuan et al., 2016; Zheng et al., 2016). On the other hand, it has also been thought that the glaciation cycles of the Quaternary have changed distribution ranges of species and generated repeated contraction-expansion processes (Hewitt, 2000). These changes are particularly common for organisms in temperate North America and Europe, where similar patterns of decreased genetic diversity and palaeoclimate-associated population fluctuations in newly established populations have been identified (Smith et al., 2022).

Although local adaptation to environments is considered to be of commonplace occurrence, detecting its occurrence can be hard and need substantial research efforts (Blanquart et al., 2012; Blanquart et al., 2013; Savolainen et al., 2013). Generally, detection of local adaptation have been based on quantitative genetic approaches that use common garden experiments and statistical genetics approaches to detect genetic differentiation for phenotypic traits (Hereford, 2009). Afterwards, quantitative genetic methods were often combined to population genetic tools to detect local adaptation (Badyaev et al., 2002; Kremer and Le Corre, 2012; Leinonen et al., 2013). More recently, advances in high-throughput sequencing technologies have made it feasible to detect genomic regions driving local adaptation (Pool et al., 2010; Hancock et al., 2011; Jones et al., 2012). Within such approaches, it has been considered that genome scan or outlier detection methods can detect adaptive differentiation without the application of common garden experiments (Storz, 2005; De Mita et al., 2013; Lotterhos and Whitlock, 2014).

*Brachymystax lenok* is a Asian endemic salmonid fish, which lives in almost all major Siberian river systems and can be used as a model for investigating paleohydrological events and climate adaptation (Froufe et al., 2008). There are generally two different morphological forms of *Brachymystax lenok*, differing mainly in the length and shape of their snouts and many external morphological and osteological characters. According to these morphological differences, this species is named as *B. lenok* and a subspecies, *B. lenok tsinlingensis* (Alekseyev et al., 2002; Xu et al., 2009). Subspecies *B. lenok* is a sharp-snout form which is distributed in Siberia and Mongolia, while *B. lenok tsinlingensis* is a blunt-snout form, occurring in China and the Korean peninsula (Froufe et al., 2008). Studies using morphology found that there are variation of morphology within each form among major basins (Alexeev et al., 1986). However, studies based on allozyme variation suggested that there is limited gene flow between the two forms (Shedko and Ginatulina, 1993). Some enzyme systems can also distinguish sharp- and blunt-snouted lenoks. However, we still lack a whole-genome scale study to comprehensive characterize the population structure and environment adaptation of *B. lenok* populations.

In this study, we performed a whole-genome sequencing (WGS) method to examine the population structure, evolutionary history and genomic signatures underlying climate adaptation in 5 *B. lenok* populations. The objectives of this study were to (i) investigate the population structure of *B. lenok* across the main distribution area in China; (ii) examine the demographic history of *B. lenok* and test whether the population dynamics has been affected by climatic oscillations during their evolution; and (iii) test whether adaptive signatures happen in the different populations, and, if so, identify the genomic regions of adaptation by investigating genome-wide genetic variation and differentiation in different populations. Our results will increase our understanding of the genetic differentiation patterns and adaptive genetic mechanisms of *B. lenok* populations during their evolution.

## Materials and methods

### Sample collection

A total of 24 specimens of *B. lenok* from 5 localities were sampled from Shanxi, Gansu, Hebei, and Jilin Province, China (Table 1). A small piece of fin from each individual was dissected out and frozen in liquid nitrogen quickly, and stored in the refrigerator at −80°C for use. As *B. lenok* is protected species, all experimental protocols were approved by the ethics committee of Fisheries station and Fisheries administration of local government.

**TABLE 1:** Information of 24 samples from 5 geographic populations of *Brachymystax lenok*.

Sampling site	Samples number	Samples ID	Coordinate	Location
Baiyun Gorge (byx)	4	Ms22; Ms26; Ms29; Ms31	E107°37′26.54″, N34°2′11.80″	Taibai, Shanxi Province
Taibai River (tbh)	5	Ms34; Ms38; Ms42; Ms46; Ms50	E107°14′16.11″, N33°50′17.21″	Taibai, Shanxi Province
Zhangjiachuan (zjc)	5	Ms60; Ms63; Ms66; Ms69; Ms72	E106°30′51.43″, N34°48′12.72″	Zhangjiachuan, Gansu Province
Yanbian (yb)	5	Ms80; Ms90; Ms100; Ms110; Ms120	E130°11′33.40″, N43°2′32.86″	Yanbian, Jilin Province
Saihan Dam (shb)	5	Ms130; Ms140; Ms150; Ms160; Ms170	E117°12′58.80″, E117°12′58.80″	Chengde, Hebei Province

### Whole-genome resequencing and SNP calling

Genomic DNA were extracted from fin samples for all 24 specimens using Puregene Tissue Core Kit A (Qiagen, MD, USA). Then, genomic libraries of insert size of 350 base pairs were constructed and genome resequencing with average of 24.2 X for each individual were performed using the Illumina X Ten sequencing platforms (Supplementary Table S1). To detect SNPs at the population level, the Illumina sequencing reads were mapped to the *Brachymystax lenok* genome with Burrows-Wheeler Alignment (BWA) tool (Li and Durbin, 2009), and polymerase chain reaction (PCR) duplicates were filtered using SAMtools (Li et al., 2009). SNP calling was performed using GATK v.4.1.2.0 (McKenna et al., 2010) with default parameters across the 24 individuals. To obtain reliable SNP, we performed a filtering step with the following set of parameters: depth ≥4, MQ ≥ 40, FS ≤ 60, QD ≥ 4, maf ≥0.05, and miss ≤0.2, according to previous study (De Mita et al., 2013).

### Phylogenetic and population structure analyses

A NJ phylogenetic tree was constructed by MEGA software (Tamura et al., 2011) with the default parameters. Population genetic structure was estimated using ADMIXTURE v1.23 software (Alexander et al., 2009) with default parameters. PCA analysis was performed for whole-genome SNPs with the program GCTA v1.24.2 (Yang et al., 2011).

### Linkage disequilibrium analysis

Linkage disequilibrium (LD) patterns for each of the 5 populations were estimated. BEAGLE 4.1 (Browning and Browning, 2007) was used for genotype phasing. We calculated the correlation coefficient (r2) between any two SNPs using VCFtools 0.1.12b (Danecek et al., 2011) with the “–hap-r2” option using the phased genotypes. Average r2 was drawed against physical distance in base pairs with R 3.3.6.

### Genomic signatures of selection and local adaptation

We performed selective analysis using filtered SNPs according to minor allele frequency (MAF: 0.05) and site integrity (INT: 0.8) (De Mita et al., 2013), and obtained highly consistent SNP sites for downstream analysis. All these analyses were conducted using PopGenome package (Pfeifer et al., 2014). The method is to calculate the population differentiation index (Fst) and nucleotide polymorphism (π) of all SNP sites in the sliding window (100 kb). Functional annotation and enrichment analysis of selective genes using COG and KEGG databases.

## Results

### Samples collection and sequencing

**FIGURE 1:**
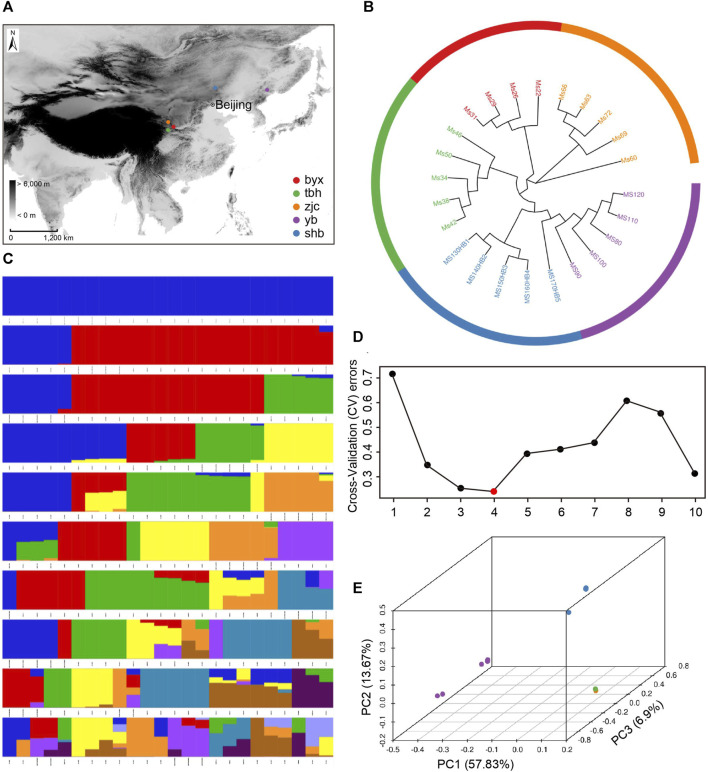
Sampling and population genetic structure of **(B)** *lenok*. **(A)** Sampling location of the five geographic populations. **(B)** Population phylogeny tree of *Brachymystax lenok* (Baiyunxia: byx, red; Zhangjiachuan: zjc, orange; Saihanba: shb, blue; Taibai River: tbh, green; Yanbian: yb, purple.). **(C)** Clustering results **(B)** lenok corresponding to each K value. **(D)** Error rate of cross verification for each K value. **(E)** Principal component analysis (PCA) of the population structure of *Brachymystax lenok*.

We sampled a total of 24 specimens of *B. lenok*from 5 localities for 4 rivers in 4 provinces, including Shanxi, Gansu, Hebei, and Jilin Province, respectively. Except specimens from Irtysh River from Xinjiang were not collected, the specimens we collected generally covered the distribution range of *B. lenok* in China. The 5 sample localities are Baiyun Gorge (byx), Taibai River (tbh), Zhangjiachuan (zjc), Saihan Dam (shb), and Yanbian (yb) (Table 1). Among these localities, Baiyun Gorge and Zhangjiachuan belong to the tributaries in the upper reaches of Weihe River, a tributary of the Yellow River and located at the north and south side of Weihe River. Baiyun Gorge is located at the north foot of Qinling Mountains. The Zhangjiachuan sampling point belongs to the Malu River of the southern extension branch of Liupan Mountain. The Taibai River is located at the southern foot of the Qinling Mountains and is a tributary of the upper reaches of the Han River, a tributary of the Yangtze River. The Saihan Dam sampling site is located in the upper reaches of Luanhe River in Chengde, Hebei. The Yanbian sampling site is located in Mijiang River in the upper reaches of Tumen River in Yanbian Prefecture, Jilin Province (Figure 1A).

The genomes of 24 individuals of *B. lenok*were resequenced (Supplementary Table S1), with an average of 97% genome coverage and 24.2 X sequencing depth for each individual based on the *Brachymystax lenok*reference genome of 2.34 Gb. The sequencing quality of these 24 individuals were higher than 96.5% for Q20 and higher than 91.8% for Q30. After filtering the low quality reads, we generated a total of 1331.6 G clean data for these 24 individuals. Using the SNP-calling strategy of the Genome Analysis Toolkit (GATK), we detected a total of 83,804,838 high quality SNPs with an average of 3,491,868 for each individual for further analysis (Supplementary Table S1). Based on these high quality whole-genome SNPs, we performed subsequent analyses, including the phylogenetic tree construction, principal components analysis (PCA), and selective analysis.

### Genetic diversity and population structure

We first evaluated the whole-genome genetic diversity for each population (Table 2; Supplementary Table S2), and identified a total of 3,351,103 SNPs for Baiyun Gorge population, 4,701,531 SNPs for Taibai River population, 4,181,141 SNPs for Zhangjiachuan population, 43,798,630 SNPs for Yanbian population, and 11,671,153 SNPs for Saihan Dam population, respectively. These SNPs from whole-genome level we identified extremely expanded the genetic diversity for *B. lenok*populations. Overall, the genetic diversity of population from sampling sites of eastern China (Yanbian and Saihan Dam) were significantly higher than those from sampling sites of middle China (Baiyun Gorge, Taibai River, and Zhangjiachuan). We then located the position of each SNP on the *B. lenok*genome, and found that most of these SNPs are located in intergenic region and intron region.

**TABLE 2:** SNP distribution in different geographical populations of *Brachymystax lenok*.

Variation type	Baiyun Gorge	Taibai river	Zhangjiachuan	Yanbian	Saihan Dam
Intergenic region	1406980	1993143	1758300	20176992	5182315
Intron region	1735103	2428700	2163179	22129556	5978386
Synonymous mutation	108065	143904	133757	839133	269810
Nonsynonymous mutation	99260	133511	123804	641771	236437
Stop codon gain	1177	1609	1454	8601	3080
Stop codon loss	518	664	647	2577	1125
Total	3351103	4701531	4181141	43798630	11671153

We then explored the structure of *B. lenok* using phylogenetic tree, principal components analysis (PCA), and ADMIXTURE analysis. The unrooted neighbour joining (NJ) tree showed that population from Baiyun Gorge, Taibai River, and Zhangjiachuan clustered together, which are separated from the clade including population from Yanbian and Saihan Dam. Each population in our dataset clustered together to form a independent clade in the phylogenetic tree, except one individual (Ms170) from Saihan Dam to be clustered within Yanbian population. This result suggested that each geographical population exists independently and lacks gene flow (Figure 1B).

The analysis of population structure can quantify the number of ancestors and infer individual genetic assignment (Figure 1C). In the admixture plot at K = 2, assuming that all the samples come from two ancestral groups, this subdivision clearly distinguishes Yanbian population from the other four populations. At K = 3, it is assumed that all samples come from three ancestral groups, namely, population from Baiyun Gorge, Taibai River and Zhangjiachuan, shared a common ancestor, and population from Saihanba and Yanbian have a independent ancestor, respectively. At K = 4, it is assumed that all samples are from four ancestral groups, of which population from Baiyun Gorge and Zhangjiachuan shared a common ancestor, and population from Taibai River, Saihanba and Yanbian have three independent ancestors. According to the cross-validation (CV) errors (Figure 1D), K = 4 shows lowest CV value. Therefore, the 5 population in our study should come from 4 ancestors, among which population from Zhangjiachuan and Baiyun Gorge, both located in the tributaries of Weihe River, share a common ancestor.

We further investigated the population structure of *B. lenok* using principal component analysis (PCA) (Figure 1E). The *B. lenok* can be divided into 3 population using the first three principal components (PC1, PC2, PC3; explained 57.83%, 13.67%, 6.9%), with the population from Baiyun Gorge, Taibai River and Zhangjiachuan forming a sub population, and population from Saihanba and Yanbian forming a independent sub population, respectively. Taken together, all these analyses demonstrated that different geographic population of *B. lenok* showed significant genetic divergence, with the eastern China population (Yanbian and Saihan Dam) divergent with the middle China population (Baiyun Gorge, Taibai River, and Zhangjiachuan).

### Linkage disequilibrium and phylogenetic analysis

**FIGURE 2:**
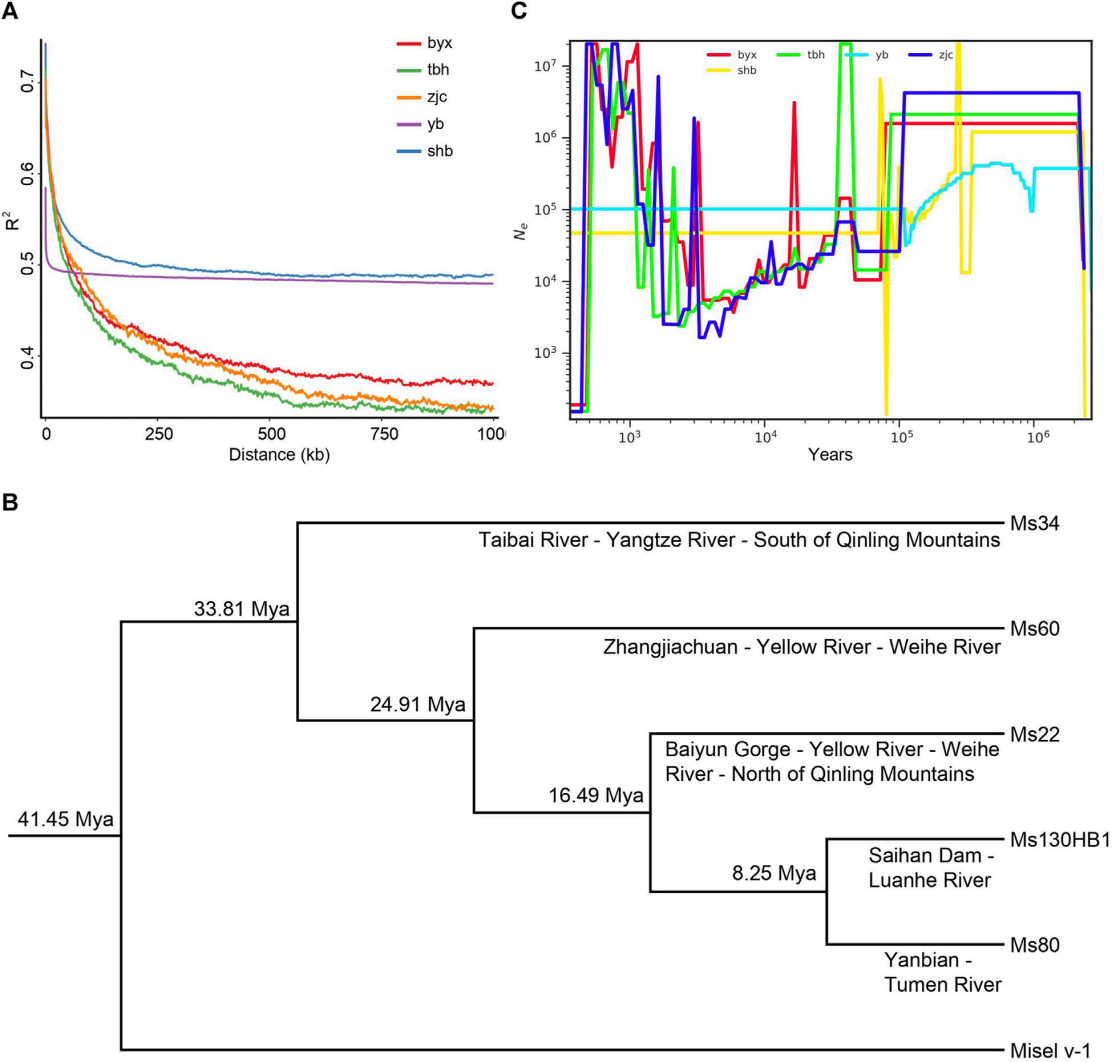
Linkage disequilibrium and population demography of **(B)** *lenok*. **(A)** Linkage disequilibrium decay distance (LDD) for five geographic populations. **(B)** Historical effective population size of **(B)** lenok, X-coordinate is time by year, Y coordinate is effective population size value. **(C)** Phylogenetic tree with divergence time of five geographical populations.

The decay of linkage disequilibrium is usually closely related to the genetic recombination probability of the population, which is measured using *R* ^2^ and stronger linkage disequilibrium with *R* ^2^ closer to 1. Our results showed that the linkage disequilibrium intensity of Baiyun Gorge, Saihanba, Zhangjiachuan and Taibai River groups are all higher than 0.7, while population from Yanbian showing the weakest of 0.59 for *R* ^2^ (Figure 2A). When the value of *R* ^2^ decrease to half, the decay distance of Saihan Dam and Yanbian group cannot be obtained. However, the decay distance of Banyun Gorge is 752 kb, the decay distance of Zhangjiachuan is 636 kb, and the decay distance of Taibai River is 472 kb, respectively. These results suggested that the probability of recombination within the population within the same physical distance is Taibai River > Zhangjiachuan > Baiyun Gorge.

We finally performed phylogenetic analysis for these 5 population of *B. lenok* (Figure 2B) using SNPs from single-copy orthologs. We filtered the SNPs, with integrity of 80%, maf 0.05, and 27,113,585 SNP sites were retained. With the Atlantic salmon as the outgroup, our results showed that the Taibai River population in the upper reaches of the Han River at the southern foot of the Qinling Mountains firstly diverged. The divergence time with the reference genome Atlantic salmon is 41.45 Mya, which belongs to the oldest population. The divergence time between the Taibai River population and the entire northern population is 33.81 Mya. The Zhangjiachuan population first diverged at 24.91 Mya. The Baiyun Gorge population diverged from the Saihanba and Yanbian populations at 16.49 Mya, while the Saihanba and Yanbian populations diverged at 8.25 Mya.

### Population demographic history

We further estimated the demographic history of *B. lenok* (Figure 2C) and found that the effective population size of the Taibai River population decreased 6 times in history. The first decrease occurred about 2.34 Mya (Million years ago), and then the effective population increased from 0.23 million to 2.12 million at 2.14 Mya. This peak period lasted until 0.08 Mya. Another peak occurred 0.04 Mya, with the number of effective populations reaching 20.33 million. In the subsequent 0.034 Mya, the number of effective populations reduced to 33,000. After that, there were several small fluctuations. The number of effective populations again expanded to 16.81 million before 684, and the number of effective populations reduced to 155 before 484, which has been the lowest in history until now. The effective population size of Baiyun Gorge increased from 20,000 to 1.58 million at 2.13 Mya, and then maintained this value for more than 2 million years. The effective population decreased to 10,000 by 0.07 Mya, expanded to 140,000 by 0.04 Mya, decreased to 8,000 about 0.02 Mya, then increased to 3.09 million about 0.017 Mya, and then decreased to 17,000 at 0.015 Mya. The highest effective population appeared at 566 years ago, reaching 20.33 million, Then, in 476 years ago, it dropped to the current 190, also the lowest value in history. The effective population size of Zhangjiachuan population shows a similar trend with that of Baiyun Gorge, with the highest value of 20.33 million, which appeared at 800 years ago and 518 years ago respectively, and the lowest value of 153, which appeared 435 years ago and has been continuing up to now. The effective population size of Yanbian population remained above 370,000 before 1 Mya, decreased to 95,000 during 0.95 Mya to 0.98 Mya, and then recovered to 200,000. It reached the highest value of 440,000 during 0.566 Mya, and then continued to decline. The effective population value decreased to 95,000 at 0.14 Mya, and the effective population size decreased to 32,000 at 0.11 Mya. The effective population value has maintained about 100,000 since 0.1 Mya. The effective population size of Saihan Dam population was 1.2 million since 0.348 Mya. The effective population size decreased to 13,000 at 0.33 Mya. Then there was another significant population expansion in between 0.27 Mya and 0.28 Mya. The effective population size reached the maximum of 20.33 million, and then began to decline. The population decreased significantly around 0.08 Mya, and the effective population size decreased to the minimum of 139. Since 0.07 Mya, the effective population value has remained at 47,000.

### Genomic signatures of selection

**FIGURE 3:**
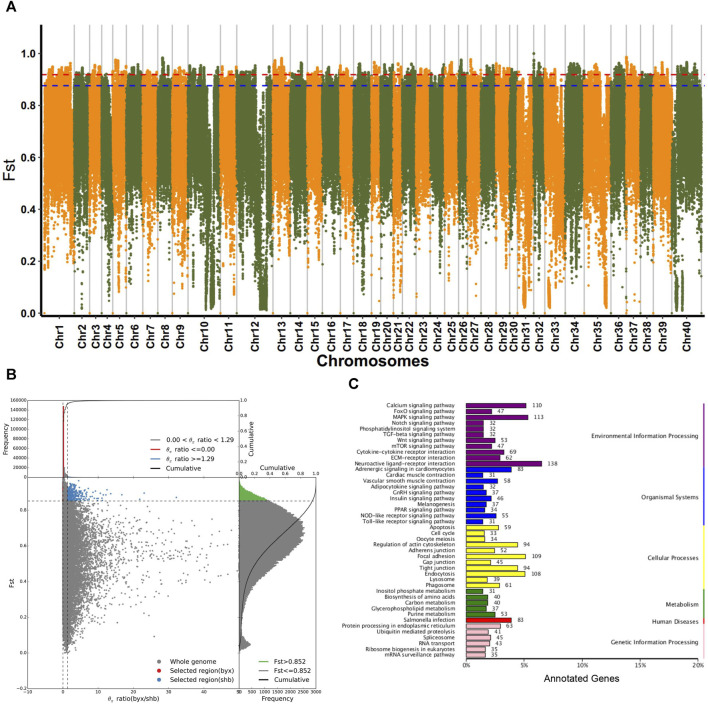
Selective analyses for population of **(B)** *lenok*. **(A)** Fst Manhatton diagram for each pairwise comparison of 5 geographic groups of *Brachymystax lenok*. **(B)** Combination of top5% and π ratio of 5 geographic groups. **(C)** KEGG annotation results of selected regions in byx population (byx vs. shb).

Considering that the 5 geographic populations of *B. lenok* live in different geographic ranges and climate environments and experienced significant genetic divergence, we then focused on the identification of genomic signatures of selection and local adaptation between these different geographic populations. We identified the selected genes using both Fst and π ratio. As shown in Figure 3A, Manhattan plot mainly identified the top1% and top5% based on the population differentiation index (Fst). By combining top5% Fst and π ratio, we identified a list of selective genes in the pairwise comparison of the 5 different geographic populations (Figure 3B; Supplementary Table S3).

Functional enrichment analyses of these selected gene showed that in terms of biological process, selected genes in Baiyun Gorge and Saihan Dam populations mainly includes cell process, biological regulation, metabolic process, stress response. The molecular functions of these selective gene are mainly enriched in the catalytic activities, regulation of molecular functions, transfer activities. COG annotation results showed that selective genes of two geographic populations mainly enriched in signal transduction mechanisms, general function prediction genes, replication recombination and repair genes. The enrichment of KEGG function showed that selective genes of the two geographical populations mainly enriched in receptor interaction pathway, MAPK signal pathway, *salmonella* infection pathway, cell adhesion pathway, and Wnt signal pathway. These results indicated that the two geographical groups were selected under the pressure of their habitat environment in the historical evolution, and the difference comparison was shown at the molecular level (Figure 3C; Supplementary Table S4).

## Discussion

The genus *Brachymystax* was firstly named by Günther in 1866, based on the population discovered in Yenisei River in 1773 by Pallas. They are mainly distributed in the rivers of China, Russia, North Korea, South Korea, Mongolia and other countries in northeast Asia, including China’s Qinling Mountains and Liupan Mountains, the upstream tributaries of the Wei River and Han River, the upstream of the Luan River in Hebei Province, the Erqis River in Xinjiang, the Ob River, Yenisei River, Lena River to the Kolema River, the upstream of the Liao River, the Tumen River, Yalu River, Heilongjiang River, Songhua River, the Han River in Korea, and the Luodong River in South Korea. There is no consensus on the classification of the genus *Brachymystax* in the above distribution areas, and many researchers thought that there is only one species (*B. lenok*) in genus *Brachymystax*. Li (1966) named the *B. lenok* in the Qinling Mountains of Shaanxi Province and Gansu Province in the upper reaches of the Weihe River as the Qinling subspecies, *Brachymystax lenok tsinlingensis* (Li, 1966). However, Ma, et al. thought that there are two forms of *B. lenok*, sharp snout and blunt snout, in Heilongjiang basin (Ma et al., 2005). Different from previous studies using mitochondria and few nuclear genes, our study is the first time to use genome-wide data to explore the origin and evolution of *B. lenok*, as well as the relationship between geological events.

The results of phylogenetic analysis and population structure showed that the five sampled geographic populations were separated from each other and lacked gene flow. It can be obviously divided into four groups according to the population structure analysis, and populations from Baiyun Gorge and Zhangjiachuan are the same ancestor group. Divergence time estimation shows that Taibaihe population diverged from other populations firstly, indicating that Taibaihe population may be the oldest population. According to the divergence time of 41.45 Mya with Atlantic salmon and the comparison with geological time, it is found that this period is in Eocene and Oligocene. Geological studies generally believes that this period (56Mya - 38Mya) was the peak period of collision between the Indian plate and the Asian plate. At that time, the environment in the middle of the Qinghai Tibet Plateau was warm and humid, and the altitude was low, which has not yet risen to today’s height. At that time, the Qinghai Tibet Plateau was still in the Tethys Sea, and the Atlantic Ocean was in the early stage of development. Therefore, *B. lenok* may have entered the southern foot of the Qinling Mountains from the Atlantic Ocean and the Tethys Sea. Later, with the uplift of the Qinghai Tibet Plateau and the demise of the Tethys Sea, it was landlocked to the freshwater environment, and began to evolve separately from the Atlantic salmon, which can also explain the distribution of the fine scale salmon in the Erqis River, the Ob River and other regions of Siberia.

Then, Zhangjiachuan population diverged from other populations at 33.81 Mya and Baiyun Gorge population was separated from Saihanba and Yanbian populations 24.91 Mya. This time was just the end of the peak of collision between the Indian plate and the Asian plate, and also the stage of rapid uplift of the Qinling Mountains. Geological studies shows that the Cenozoic tectonic uplift of the Qinling orogenic belt was based on the late Cretaceous (99.6 Mya) tectonic uplift, and began to rapidly uplift in the middle Eocene (55.8 Mya) - Oligocene (33.9 Mya), while the early Pleistocene (1.806 Mya) was the strongest period of neotectonics, Moreover, various geological studies and events show that Qinling Mountains are still rising relative to Weihe Basin. Therefore, the divergence of Zhangjiachuan and Baiyunxia populations may be related to the uplift of the Qinling Mountains. The possible explanation for why the Baiyun Gorge population could not separate from the Saihanba and Yanbian populations until 16.49 Mya is that they still had gene flow during 24.91 Mya −16.49 Mya. Unlike plants, fish cannot migrate through wind, artificial transplantation, etc., they must rely on rivers in waters. Therefore, it suggested that the Baiyun Gorge population can still have gene flow with the Saihanba and Yanbian populations through rivers or oceans after no gene flow with Zhangjiachuan population. The only river supporting this gene flow is the Weihe River, so the Weihe River may have been open to the sea at that time. As for the reason for the divergence between Baiyun Gorge and Zhangjiachuan populations, further research is needed by combining geological events. In addition, the divergence between Saihanba and Yanbian populations, as well as the divergence of populations in northeast basins, Russia and South Korea, need to increase sampling points and sample size for further research.

According to the analysis of genetic evolution, the population in the Qinling Mountains is older than that in Yanbian, but its morphology is just the opposite. In the field sampling survey, it is also found that the morphology of *B. lenok* in the northeast region is more rough and clear, showing more primitive characteristics. On the contrary, the morphology of *B. lenok* in the Qinling Mountains is more rounded and softer, showing more characteristics of environmental selection. This suggests that in the evolution process of *B. lenok*, the population in the Qinling Mountains has been subjected to greater environmental selection pressure due to the land seal. In order to adapt to the habitat environment of the mountains, streams and rivers in the Qinling Mountains, a greater epigenetic change has taken place. The population in the northeast has better river habitat, less environmental selection pressure, and the habitat conditions have little impact on its epigenetic. Therefore, the population in the northeast has retained more original characteristics in the evolution process. However, the genetic basis of these phenotypic difference needs further investigation in future.

However, in this study, we only collected data from a limited number of individuals for population genomic analyses due to the protected status of this fish species, which may have a bias on our results. The limitation of a low number of samples for population genomic analyses is multifaceted and critical to acknowledge in genetic research. A reduced sample size significantly diminishes the statistical power of analyses, rendering it challenging to accurately detect subtle genetic variations or associations within populations. This limitation not only impedes the identification of meaningful patterns or trends but also hampers the generalizability of findings to larger population groups. Additionally, a small sample size may introduce sampling biases, leading to results that are not representative of the broader population. Consequently, while population genomic analyses offer invaluable insights into genetic diversity and evolutionary dynamics, the constraints imposed by a limited sample size underscore the need for caution in interpreting findings and highlight the importance of expanding sample sizes to ensure robust and reliable conclusions in genetic studies.

## Conclusion

In this study, we analyzed the phylogenetic relationship, population structure, gene flow and genetic diversity of five geographic populations of *B. lenok* by using whole genome SNPs. Our results showed widespread genetic divergence in *B. lenok* from different geographic populations, and confirmed that their current distribution pattern are driven by geographical isolation and changes in oceans and rivers. We also identified the genes evolved to adapt the different environments, including the stress adaptation. Overall, our study provides new insights into the origin, evolution, and adaptation of *B. lenok*.

## Data Availability

The datasets presented in this study can be found in online repositories. The SRA number is PRJNA946352.
